# Towards the Definition of Radiomic Features and Clinical Indices to Enhance the Diagnosis of Clinically Significant Cancers in PI-RADS 4 and 5 Lesions

**DOI:** 10.3390/cancers15204963

**Published:** 2023-10-12

**Authors:** Pietro Andrea Bonaffini, Elisabetta De Bernardi, Andrea Corsi, Paolo Niccolò Franco, Dario Nicoletta, Riccardo Muglia, Giovanna Perugini, Marco Roscigno, Mariaelena Occhipinti, Luigi Filippo Da Pozzo, Sandro Sironi

**Affiliations:** 1 Department of Radiology, ASST Papa Giovanni XXIII, Piazza OMS, 24127 Bergamo, BG, Italy; 2 School of Medicine, University of Milano-Bicocca, Piazza dell’Ateneo Nuovo, 1, 20126 Milan, MI, Italy; 3 Medicine and Surgery Department, Via Cadore, 48, 20900 Monza, MB, Italy; 4 Interdepartmental Research Centre Bicocca Bioinformatics Biostatistics and Bioimaging Centre-B4, University of Milano-Bicocca, Via Follereau 3, 20854 Vedano al Lambro, MB, Italy; 5 Department of Urology, ASST Papa Giovanni XXIII, Piazza OMS, 1, 24127 Bergamo, BG, Italy; 6 Radiomics, Boulevard Patience et Beaujonc 3, 4000 Liège, Belgium

**Keywords:** PI-RADS v.2.1, prostate cancer, clinically significant cancers, multiparametric prostate MRI, radiomics

## Abstract

**Simple Summary:**

Lesions scored as PI-RADS 4 and 5 may include false positives and tools aimed to reduce them are highly needed. MRI texture analysis of standard sequences seems to improve the detection of clinically significant cancer (csPC) in PI-RADS 4/5 lesions. Multivariate models considering both clinical and radiomic features achieved promising diagnostic values.

**Abstract: Objectives:**

Lesions classified as PI-RADS 4/5, according to the Prostate Imaging–Reporting and Data System (PI-RADS) guidelines, may include false positives. This study aims to identify promising radiomic features that may support the detection of clinically significant tumours among PI-RADS 4/5 lesions on MRI. **Methods:** Patients undergoing a 3T magnet multiparametric MRI (mpMRI) for clinical suspicion of prostate cancer (PC) or active surveillance were retrospectively enrolled. Pathological results utilizing MRI-targeted biopsy specimens were considered the ground truth. Clinical (age, PSA, PSA *density*) and MRI parameters (prostate volume, mean apparent diffusion coefficient/ADC) were collected. Lesions were manually contoured on axial T2-weighted images and ADC maps. Radiomic features were extracted with *Pyradiomics*. Clinical and radiomic features best correlating with histopathological results were selected. Diagnostic values were assessed on validation samples. **Results:** The final cohort included 99 patients (mean age, 69.2 ± 6.8 years) and 111 PI-RADS 4/5 lesions. At pathology, 79 lesions (71%) were identified as clinically significant cancers (Gleason score ≥ 7). Radiomic, clinical, and MRI features best correlating with histopathology were selected. The best predictive clinical and radiomic multivariate model showed the following diagnostic values: sensitivity, 79%; specificity, 80%; positive predictive value (PPV), 91%; negative predictive value (NPV), 63%; accuracy, 79%. A radiomic multivariate model based exclusively on peripheral zone lesions showed more promising values: sensitivity, 86%; specificity, 80%; PPV, 93%; NPV, 70%; accuracy, 84%. **Conclusions:** Radiomic MRI feature analysis can potentially improve the accuracy of mpMRI in discriminating between clinically significant cancers in PI-RADS 4 and 5 lesions.

## 1. Introduction

Prostate cancer (PC) is the most frequently diagnosed cancer among adult men, and its incidence is increasing worldwide [1,2]. The risk of PC is related to age, family history, ethnicity, and prostate-specific antigen (PSA) levels [3].

Multiparametric magnetic resonance imaging (mpMRI) is considered the imaging gold standard for PC diagnosis, staging, and follow-up during active surveillance [4,5]. The Prostate Imaging–Reporting and Data System (PI-RADS), version 2.1, represents the cornerstone of prostate MRI reading [6]. PI-RADS criteria require the interpretation of radiologists to score prostate lesions into five categories that represent their probability of being clinically significant cancer (csPC). csPC is defined as tumours showing a Gleason score (GS) equal to or greater than seven at histopathological analysis [7,8]. In the case of PI-RADS 4- or 5-rated lesions, the occurrence of a csPC is likely or highly likely, respectively.

However, this reporting system does not supply numerical data on expected cancer detection rates for each category. In this regard, a recent systematic review reported that 52% of PI-RADS 4 and 89% of PI-RADS 5 lesions represent csPC [9]. Another review, based on PI-RADS version 2 criteria, revealed positive predictive values (PPVs) of 40% and 69% for the PI-RADS 4 and 5 categories, respectively [10]. In particular, transition zone (TZ) lesions show a lower PPV compared with peripheral zone (PZ) ones because of TZ heterogeneity, which makes PC detection more difficult [11,12]. False-positive PI-RADS 4 and 5 lesions are reported to correspond to inflammatory, stromal, glandular, and vascular alterations beside clinically non-significant tumours [13]. Even if the PI-RADS does not provide specific management recommendations for each category, the standard clinical practice is to refer patients with PI-RADS 4 or 5 lesions to biopsy [14]. However, prostate biopsies are cost-intensive techniques, require specific equipment and trained operators, and are associated with patient discomfort and potential complications, such as haematuria, rectal bleeding, haematospermia, and sepsis [15]. For this reason, further improvements in non-invasive cancer detection rates may help reduce unnecessary biopsies.

In recent years, there has been an ever-growing interest in the quantitative analysis of imaging data. Radiomics is an emerging scientific field that aims to extract and analyse quantitative information related to the properties of tissues and lesions from medical images, assuming that they contain more data than an expert radiologist can discern [16]. This information is extracted as features from regions or volumes of interest (ROIs or VOIs) and can be classified as shape, first-order, or higher-order features. Radiomics has already demonstrated promising results in PC detection, aggressiveness assessment, and treatment evaluation [17].

On this basis, the aim of the present study is to identify promising radiomic features that, combined with routine clinical and imaging parameters, may support the detection of csPC in PI-RADS 4 and 5 lesions in prostate mpMRI studies.

## 2. Materials and Methods

This retrospective study was conducted in accordance with the Declaration of Helsinki. Clinical and radiological data were anonymized. The study was approved by the local Ethics Committee (protocol code “*mp-MR e Sorveglianza Attiva*”—07/06/2018).

### 2.1. Patient Sample

This study included consecutive patients who underwent a pelvic mpMRI between June 2016 and March 2021 performed at our institution for suspected PC or during active surveillance. The inclusion criteria were as follows: (1) a lesion scored as PI-RADS 4 or 5 according to PI-RADS v2.1 guidelines; (2) an mpMRI scan performed before or at least eight weeks after the biopsy; (3) available histopathological data from MRI-targeted biopsies. Exclusion criteria were as follows: (1) patients with absolute contraindications to MRI (i.e., pacemakers/defibrillator carriers) or MRI performed in an outside institution; (2) poor image quality on the T2-weighted (T2W) and/or diffusion-weighted imaging (DWI) sequences; (3) no definite final histopathological data available.

Data regarding patients’ demographic, laboratory, pathological, and conventional MRI parameters were collected: patients’ age; the most recent serological value of PSA (ng/mL); PSA density (total PSA/prostate volume ratio); final histopathological results and GS assessment; prostate gland volume (cc); and mean apparent diffusion coefficient (ADC) value (mm^2^/s), measured with a 2D ROI delimitating the largest possible area within the lesion.

### 2.2. Definition of the Reference Standard

Histopathological results from targeted fusion biopsies of lesions scored as PI-RADS 4 and 5 served as the ground truth. Biopsies were performed by a highly experienced operator (total experience > 500 biopsies) through transrectal access and the fusion technique with a reference MRI, a MyLabClassC ultrasound machine, and a virtual navigator fusion system (Esaote S.p.A., Genova, Italy), using an end-fire endorectal probe. Each patient underwent a targeted biopsy of PI-RADS 4–5 lesions (4 cores), and additional systematic biopsies (12–16 cores, according to the following prostate volumes: ≤60 mL vs. >60 mL) were performed. Histopathological confirmatory reports were acquired from our institution’s Department of Pathology medical records. GS was assigned according to the 2005 International Society of Urological Pathology (ISUP) recommendations [18,19]. csPC was defined as an ISUP Grade Group of 2 (GS 3 + 4 = 7). PC-positive biopsies were evaluated in conformity with the ISUP 2014 consensus Gleason Grade Group system [19].

### 2.3. MRI Acquisition

All patients underwent a standardized departmental MRI protocol on a 3T scanner (Discovery MR750w, General Electric Healthcare, Chicago, IL, USA) using a 16-channel pelvic anterior array coil (General Electric Healthcare, Chicago, IL, USA). The examination was performed before or at least eight weeks after the biopsy in order to reduce biopsy-related impacts on MRI reading and PI-RADS score assessment, such as haemorrhage or inflammation [20]. The mpMRI protocol included the following: (1) T2-weighted (T2W) images on axial, sagittal, and coronal planes; (2) DWI on axial plane with automatically generated ADC maps; (3) T1-weighted (T1W) sequences on axial planes with and without fat saturation, acquired before and after intravenous gadolinium-based contrast. MRI protocol technical details are listed in Table 1. An anti-peristaltic agent (hyoscine butyl bromide, 20 mg/mL; Boehringer Ingelheim, Ingelheim, Germany) was intravenously administrated prior to the scan unless contraindicated. To reduce rectal gas-induced artefacts, adequate bowel preparation with a self-administered cleansing enema was required before imaging.

### 2.4. Image Analysis and Lesion Segmentation

A radiologist with >5 years of experience in prostate MRI reading, blinded to the pathological data, re-evaluated the MR examinations and scored all the lesions strictly in compliance with PI-RADS v2.1 guidelines.

**Figure 1:**
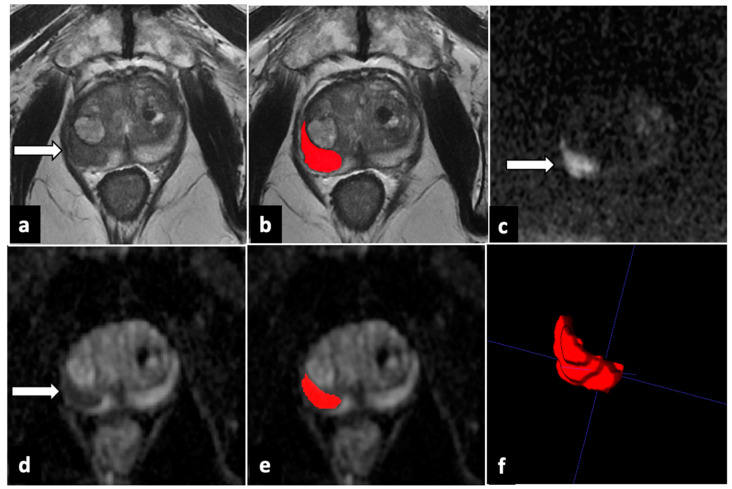
ROI delineation and generated 3D VOI in a peripheral zone lesion (*white arrows*). In the axial T2-weighted image (**a**), a markedly hypointense lesion exceeding 15 mm in length is observed in the anterior/posterior-lateral right peripheral zone of prostate midportion. The lesion showed high signal intensity in DWI (**c**) and low signal intensity in the ADC map (**d**). The lesion was scored as PI-RADS 5 and manually contoured on T2-weighted and diffusion-weighted images (**b**,**e**). A 3D VOI of the whole lesion (**f**) was also generated. ADC: apparent diffusion coefficient; DWI: diffusion-weighted imaging; PI-RADS: Prostate Imaging–Reporting and Data System; ROI: region of interest; 3D VOI: three-dimensional volume of interest. Red part shows the contouring and final volume obtained from the lesion.

**Figure 2:**
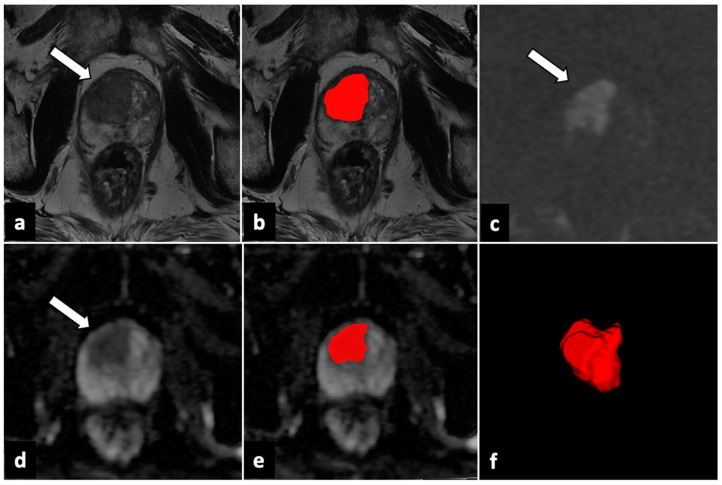
ROI delineation and generated 3D VOI in a transitional zone lesion. In the right transitional zone of the gland apex, an ill-defined lesion with a major diameter >15 mm is observed (*white arrows*). The lesion shows a moderately low signal in the T2W sequence (**a**), a high signal in DWI (**c**), and signal loss in the ADC map (**d**), consistent with a PI-RADS 5 category. It was segmented with T2W and ADC (**b**,**e**), and a 3D VOI of the lesion (**f**) was constructed. ADC: apparent diffusion coefficient; DWI: diffusion-weighted imaging; ROI: region of interest; PI-RADS: Prostate Imaging–Reporting and Data System; T2W: T2-weighted; 3D VOI: three-dimensional volume of interest. Red part shows the contouring and final volume obtained from the lesion.

For each study with at least one lesion rated as PI-RADS 4 or 5, T2W, b-values of 2000 s/mm^2^ DWI, and ADC maps were downloaded from the Picture Archiving and Communication System (PACS) in anonymized Digital Imaging and Communication in Medicine (DICOM) format. DICOMs were successively imported in segmentation software, ITK-SNAP 3.8.0 (PICSL, University of Pennsylvania, Philadelphia, PA, USA). All the VOIs of every PI-RADS 4 or 5 lesion were manually drawn slice by slice on T2W sequences and ADC maps by a junior radiologist (3 years of experience in prostate mpMRI reading) and then validated by a board-certified radiologist (>5 years of experience) (Figure 1 and Figure 2). Moreover, an additional VOI was delineated in the healthy prostate peripheral zone (PZ), avoiding chronic prostatitis changes, in order to normalize signal intensity for each patient.

### 2.5. Radiomic Feature Extraction, Selection, and Analysis

Before radiomic processing, all the images were corrected for magnetic field inhomogeneity (algorithm N4, 3D Slicer 4.13.0, http://www.slicer.org, accessed on 28 December 2021). In addition, T2W signal intensities within the lesion VOIs were normalized to reduce signal variations across subjects by ranging them at the mean intensity value obtained through the aforementioned additional VOI [21]. T2W and ADC VOIs were resampled on 0.4 × 0.4 × 3.0 mm^3^ and a 0.8 × 0.8 × 3.0 mm^3^ voxel grids, respectively, through b-spline interpolation.

The lesions’ radiomic features were extracted using the open-source PyRadiomics package (https://pyradiomics.readthedocs.io/) with original images (32 bin quantization), and images filtered with low-pass (LLL) and high-pass (HHH) coif1 wavelet filters of the decompositions (8 bin quantization for T2 and 16 bin quantization for ADC). A total of 586 features per lesion (293 in T2W and 293 in ADC images) were extracted. Three different analyses were computed considering (1) both PZ and TZ PI-RADS 4 and 5 lesions; (2) only PZ lesions; and (3) only TZ lesions.

Radiomic, demographic, laboratory, and conventional MRI indices more robustly related to pathological results were selected by randomly dividing the lesions into 5 groups 100 times (maintaining the clinically significant and non-significant tumours’ balance). In each of the 500 feature selection trials, the Wilcoxon non-parametric test assessed the univariate association between features and pathological findings. Spearman rank correlations were used to evaluate the association between textural features, and features correlated to each other were discarded (Spearman rank > 0.5). The Benjamini–Hochberg method for multiple testing was applied (false discovery rate of 0.05 for the first two analyses; and 0.1 for the TZ lesion-based analysis). Features selected in more than 20% of the 500 trials (10% for the TZ lesion-based analysis) were chosen.

### 2.6. Statistical Analysis

Univariate and multivariate models’ definition and assessment analyses were performed using a 5-fold stratified cross-validation scheme. In particular, patients were randomly divided 100 times into 5 groups, maintaining the balance of clinically significant and non-significant cancer. Univariate models were defined by choosing thresholds that maximized the Youden index in training sets and were assessed in terms of sensitivity and specificity in test sets. For each selected feature, the mean and standard deviation of sensitivity, specificity, PPV, NPV, and accuracy in the 500 trials were reported. Six classification models were considered for multivariate analysis: linear discriminant, linear, quadratic, cubic support vector machine (SVM), classification tree, and K-nearest neighbours (KNN). All possible feature combinations from the selected feature pool were evaluated as classification model inputs. Models and optimal thresholds were identified in the training sets and assessed in terms of sensitivity, specificity, PPV, NPV, and accuracy in the 500 validation sets. All the analyses were performed in Matlab (R2022b, https://it.mathworks.com/products/matlab.html).

## 3. Results

### 3.1. Patient and Lesion Characteristics

**Figure 3:**
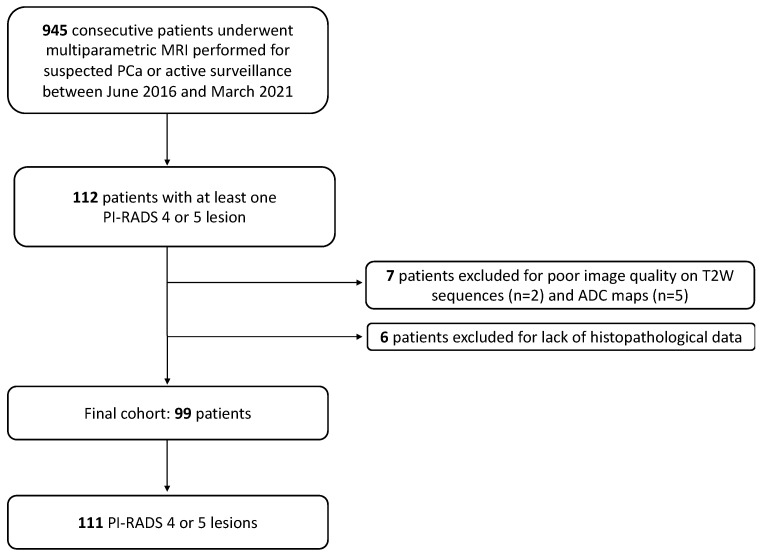
Study cohort flowchart.

Overall, 945 consecutive patients underwent a prostate mpMRI for suspected PC or active surveillance during the aforementioned study period. In this cohort, 112 patients (11.8%) were diagnosed with at least one PI-RADS 4 or 5 lesion. Thirteen patients were excluded because of poor image quality of the T2W sequence (*n* = 2) and ADC maps (*n* = 5) or because they lacked histopathological data (*n* = 6). Therefore, the final cohort of the study included 99 patients (median age 69 years, IQR 59–79). They were diagnosed with the following: one PI-RADS 4 or 5 lesion in 89 patients; two lesions in 8 patients; three in 2 patients. Thus, 111 lesions (1.12 lesions per patient) were finally scored as PI-RADS 4 or 5 and analysed. A study flowchart showing patient inclusion and exclusion is presented in Figure 3.

In total, 74 lesions (66.7%) were PI-RADS 4 (68 [91.8%] in the PZ and 6 [8.1%] in the TZ), while 37 (33.3%) were PI-RADS 5 (18 [48.7%] in the PZ and 19 [51.3%] in the TZ). At histopathological analysis, 79 lesions (71%) consisted of csPC. Table 2 summarizes the demographic, biochemical, and histopathologic characteristics of the included patients and lesions.

### 3.2. Selected Features and Univariate Model Performance

The clinical and radiomic features that were more often selected as the best correlations with the pathological results throughout the 500 selection trials are listed in Table 3. When considering both PZ and TZ lesions, PSA density, two radiomic features from the T2 images, and three from the ADC maps were selected. By analysing only PZ lesions, PSA density, the three radiomic features from the T2 images, and the two from the ADC map analysis met the selection criteria. Conversely, only one feature extracted from ADC images was chosen when considering only TZ lesions.

The performances of the univariate models’ assessment on the 500-validation trials are summarized in Table 4. A model based on an ADC feature (ADC-original_glcm_ClusterShade) resulted in the most accurate model (sensitivity: 81%; specificity: 62%; PPV: 84%; NPV: 58%; accuracy: 76%) for identifying csPC in both PZ and TZ lesions. The best univariate models for PZ lesions and TZ lesions achieved a mean sensitivity, specificity, PVV, NPV, and accuracy of 82%, 51%, 83%, 54%, and 74% and 80%, 70%, 82%, 74%, and 76%, respectively.

### 3.3. Clinical and Radiomic Multivariate Model Performance

The results obtained using the best multivariate models considering both clinical and radiomic parameters are presented in Table 5. The best ignorant zone multivariate model combined one clinical (PSA *density*) and three radiomic (two from T2 and one from ADC imaging) features in input and showed a sensitivity of 79%, a specificity of 80%, a PPV of 91%, a PPN of 63, and an accuracy of 79% in the 500 test trials.

The most accurate multivariate model for the PZ-only lesions was based on radiomic features (two from T2 and one from ADC imaging) and achieved a sensitivity of 86%, a specificity of 80%, a PPV of 93%, a PPN of 70, and an accuracy of 84%. When evaluating only TZ lesions, no multivariate model outperformed the aforementioned univariate model.

## 4. Discussion

Prostate cancer management protocols require patients classified as PI-RADS 4/5 to undergo a biopsy [14]. However, according to a recent systematic review, at biopsy, 48% of PI-RADS 4 lesions and 11% of PI-RADS 5 lesions are not csPC [9]. If we divide the analysis by zones, these percentages are likely to be lower in PZ and higher in TZ, where tissue heterogeneity is greater, and therefore, imaging detectability is more complicated. In our dataset, 28% of PZ PI-RADS 4, 17% of PZ PI-RADS 5, 50% of TZ PI-RADS 4, and 37% of TZ PI-RADS 5 lesions resulted in non-csPC at biopsy.

In the efforts to improve standard clinical care quality, several studies over the last decade have investigated the potential use of radiomics derived from mpMRI findings for PC detection, diagnosis, grading, aggressiveness assessment, and treatment evaluation [18,22]. Notably, the distinction between clinically significant (GS equal or greater than seven) and non-clinically significant (GS equal to six) PC represents an encouraging field of application for texture analysis, on the assumption that it may help reduce needless biopsies [23,24,25,26].

In this work, we explored the putative role of mpMRI radiomics in downgrading non-csPC PI-RADS 4/5 lesions. Many authors have focused on mpMRI radiomics to upgrade PI-RADS 3 lesions [26,27,28]. Nevertheless, to the best of our knowledge, only two studies have tried to apply MRI texture feature analysis to distinguish csPC in lesions scored as PI-RADS 4 and 5. In the first one, the authors proposed a downgrading algorithm for PI-RADS ≥4 lesions based on first-order ADC features and morphological T2 features. In their dataset, this approach increased the specificity for csPC from 50% to 72%, reducing sensitivity from 88% to 85%, corresponding to an overall 16% accuracy improvement [25]. Concerning the present work, we tried to introduce three improvements by (1) adding clinical features to the pool of the putative features; (2) computing not only first-order and morphological features but also higher-order features (through an IBSI-compliant tool); and (3) trying to separate PZ and TZ lesions analysis. Moreover, Ma et al. recently compared the performance of two different machine learning classification models constructed using data from a cohort of 103 PI-RADS 4/5-scored lesions, grouping them in biopsy-proven PC (*n* = 83) and benign prostate hyperplasia (BPH) cases (*n* = 20). The first model was based on radiomic features extracted from ADC images, while the second model was built using clinical indicators, such as age, PSA, and free PSA. The classifier model based on texture features showed a superior performance (AUC value = 0.936) compared with the clinical indicator model (AUC value = 0.860) [29].

Few studies evaluated prostate texture features depending on its zonal anatomy. Laschkar and colleagues demonstrated that age-related physiological zonal modifications resulted in textural changes, especially regarding the TZ [30]. Moreover, Ginsburg et al. showed that a PZ-specific classifier detected PC in the PZ with significantly higher accuracy (AUC = 0.61–0.71) compared with a zone-independent classifier trained to detect cancer throughout the entire prostate (*p* < 0.05) [31]. In a recently published paper, the authors performed an analysis based on TZ lesions, whose diagnosis is usually more difficult given the many overlapping features between cancers and stromal BPH nodules [12]. The results demonstrated that models based on quantitative ADC, shape, and texture analysis were highly accurate for diagnosing TZ PC [32].

In the global dataset, where PI-RADS v.2.1 globally has a PPV of 71% in detecting csPC, an ignorant zone clinical–radiomic model relying on PSA density in the same two T2 features contained in the PZ model and an additional ADC feature (ADC-wavelet-LLL_glrlm_LongRunHighGrayLevelEmphasis) obtained a sensitivity of 79% ± 10%, a specificity of 80% ± 15%, and a PPV of 91% ± 6%, corresponding to a mean accuracy in csPC detection of 79%.

As to our dataset of 86 PZ lesions, where PI-RADS v.2.1 globally has a PPV of 74% in detecting csPC, a radiomic model relying on two T2 heterogeneity features (T2-wavelet-HHH_glszm_GrayLevelVariance, T2-wavelet-LLL_glszm_GrayLevelVariance) and an ADC texture feature (ADC-wavelet-LLL_gldm_HighGrayLevelEmphasis) obtained a sensitivity of 86% ± 12%, a specificity of 80% ± 19%, and a PPV of 93% ± 6%, corresponding to a mean accuracy in csPC detection of 84%.

In the dataset of 25 TZ lesions, where PI-RADS v.2.1 globally has a PPV in detecting csPC of 60%, an ADC texture feature (ADC-wavelet-LLL_glcm_ClusterShade) obtained a sensitivity of 80% ± 23%, a specificity of 70% ± 30%, and a PPV of 82% ± 19%, corresponding to a mean accuracy in csPC detection of 76%.

This study has several limitations. First, the sample size was relatively limited; consequently, validating the results with an independent internal verification dataset was impossible. Moreover, the small number of patients may have resulted in the overfitting of the models. Second, the study’s retrospective and monocentric nature must be considered. Furthermore, it can be argued that the gold standard for MRI findings evaluation should be the entire pathology drawn from radical prostatectomy and not target biopsy cores. However, less than half of our patients underwent radical prostatectomy, making it impossible to perform a reliable statistical analysis with even fewer patients.

The outcomes of this study should be validated in the future using data from multicentric and prospective investigations. Third, the inhomogeneous distribution of PZ and TZ lesions limited the analysis of the eventual different features of lesions arising from different prostate zones. Finally, manual segmentation is subject to intrinsic intra- and inter-observer variability.

## 5. Conclusions

MRI radiomic features based on axial T2-weighted imaging and ADC maps can potentially support radiologists’ subjective detection of clinically significant cancer in lesions scored as PI-RADS 4 or 5 based on PI-RADS v2.1 criteria. Features identified as correlated with clinically significant cancers in the prostate zone were also different from those identified for transitional zone lesions. Prospective tests on broader samples are needed to validate this study’s results.

## Figures and Tables

**Table 1:** Dedicated prostate MRI protocol details. Examinations were performed using a 3T magnet.

Sequence	Plane	TR/TE (ms)	FOV (mm)	Slice Thickness (mm)	Gap (mm)
T2W SSFSE	axial	3290/90	320 × 240	4	0.4
T2W FRFSE	axial	7480/150	220 × 220	3	0
T2W FRFSE	sagittal	7861/150	220 × 220	3	0
T2W FRFSE	coronal	6583/150	220 × 220	3	0
T1W SSFSE	axial	620/10	512 × 256	4	0.4
T1W 3D FSPGR (DCE) Fat-saturated	axial	5.3/2.2	380 × 350	3	0
DWI (b-values: 100, 1000, 2000 s/mm^2^)	axial	7775/91	120 × 120	3	0

DCE: dynamic contrast enhancement; DWI: diffusion-weighted imaging; FOV: field of view; FRFSE: fast relaxation fast spin echo; FSPGR: fast relaxation fast spin echo; SSFSE: single-shot fast spin echo; TE: time of echo; TR: repetition time; T1W: T1-weighted; T2W: T2-weighted.

**Table 2:** Characteristics of patients and lesions included in the final analysis.

Patients (*n* = 99)			
Age (y, IQR)	69 (59–79)		
PSA (ng/mL) mean ± SD	7.9 ± 4.8		
PSA density (ng/mL^2^) mean ± SD	0.18 ± 0.15		
Prostate volume (mL) mean ± SD	51.6 ± 27.3		
**Lesions (*n* = 111)**			
ADC value (10^−6^ mm^2^/s) mean ± SD	653 ± 223		
		**Positive at biopsy (n, %)**	**Negative at biopsy (n, %)**
**Overall (*n*, %)**	111 (100)	79 (71.2)	32 (28.8)
**PI-RADS 4 (*n*, %)**	74 (66.7)	52 (70.3)	22 (29.7)
Peripheral zone (*n*, %)	68 (91.9)	49 (72)	19 (28)
Transition zone (*n*, %)	6 (8.1)	3 (50)	3 (50)
**PI-RADS 5 (*n*, %)**	37 (33)	27 (73)	10 (37)
Peripheral zone (*n*, %)	18 (48.7)	15 (83)	3 (17)
Transition zone (*n*, %)	19 (51.3)	12 (63)	7 (37)
**ISUP prostate cancer grade group (*n*, %)**			
**1** (GS ≤ 6)	32 (28.8)		
**2** (GS = 3 + 4)	46 (41.5)		
**3** (GS = 4 + 3)	20 (18.0)		
**4** (GS = 8)	11 (9.9)		
**5** (GS ≥ 9)	2 (1.8)		

ADC: apparent diffusion coefficient; GS: Gleason score; ISUP: International Society of Urological Pathology; IQR: interquartile range; PI-RADS: Prostate Imaging–Reporting and Data System; PSA: prostate-specific antigen.

**Table 3:** Selected clinical and radiomic features. For the analysis based on both peripheral (PZ) and transitional (TZ) lesions and the PZ-based analysis, features selected in more than 20% of the 500 tests were chosen. For the TZ-based analysis, features selected in more than 10% of the 500 tests were chosen.

Features	% Choices
**Peripheral and transitional zone**	
PSA density	56%
T2-wavelet-HHH_glszm_GrayLevelVariance	51%
T2-wavelet-LLL_glszm_GrayLevelVariance	21%
ADC-original_glcm_ClusterShade	24%
ADC-wavelet-HHH_firstorder_Minimum	31%
ADC-wavelet-LLL_glrlm_LongRunHighGrayLevelEmphasis	26%
**Peripheral zone**	
PSA density	21%
T2-wavelet-HHH_glszm_GrayLevelVariance	41%
T2-wavelet-HHH_glszm_LowGrayLevelZoneEmphasis	36%
T2-wavelet-LLL_glszm_GrayLevelVariance	80%
ADC-original_glrlm_ShortRunHighGrayLevelEmphasis	27%
ADC-wavelet-LLL_gldm_HighGrayLevelEmphasis	23%
**Transition zone**	
ADC-wavelet-LLL_glcm_ClusterShade	10%

ADC: apparent diffusion coefficient; PSA: prostate-specific antigen.

**Table 4:** Univariate model performance for overall, PZ-based, and TZ-based analyses.

Features	Sensitivity (% ± SD)	Specificity (% ± SD)	PPV (% ± SD)	NPV (% ± SD)	Accuracy (%)
**Peripheral and transitional zone**					
PSA density	49 ± 12	91 ± 11	93 ± 8	43 ± 7	61
T2-wavelet-HHH_glszm_GrayLevelVariance	74 ± 14	52 ± 20	80 ± 7	47 ± 17	68
T2-wavelet-LLL_glszm_GrayLevelVariance	64 ± 11	63 ± 19	82 ± 8	42 ± 10	64
ADC-original_glcm_ClusterShade	81 ± 10	62 ± 18	84 ± 7	58 ± 16	76
ADC-wavelet-HHH_firstorder_Minimum	31 ± 30	71 ± 22	44 ± 37	31 ± 10	42
ADC-wavelet-LLL_glrlm_LongRunHighGrayLevelEmphasis	49 ± 23	57 ± 30	77 ± 13	31 ± 11	51
**Peripheral zone**					
PSA density	46 ± 14	89 ± 18	94 ± 10	36 ± 9	57
T2-wavelet-HHH_glszm_GrayLevelVariance	70 ± 15	57 ± 25	83 ± 9	41 ± 17	67
T2-wavelet-HHH_glszm_LowGrayLevelZoneEmphasis	64 ± 17	61 ± 25	84 ± 9	38 ± 15	63
T2-wavelet-LLL_glszm_GrayLevelVariance	67 ± 12	81 ± 18	92 ± 8	47 ± 12	70
ADC-original_glrlm_ShortRunHighGrayLevelEmphasis	72 ± 12	68 ± 24	87 ± 8	46 ± 15	71
ADC-wavelet-LLL_gldm_HighGrayLevelEmphasis	82 ± 14	51 ± 23	83 ± 7	54 ± 24	74
**Transition zone**					
ADC-wavelet-LLL_glcm_ClusterShade	80 ± 23	70 ± 30	82 ± 19	74 ± 29	76

ADC: apparent diffusion coefficient; NPV: negative predictive value; PPV: positive predictive value; PSA: prostate-specific antigen; SD: standard deviation.

**Table 5:** Multivariate model performance for overall and PZ-based analyses.

Features	Sensitivity (% ± SD)	Specificity (% ± SD)	PPV (% ± SD)	NPV (% ± SD)	Accuracy (%)
**Multivariate model for peripheral and transitional zone**					
PSA density	79 ± 10	80 ± 15	91 ± 6	63 ± 13	79
T2-wavelet-HHH_glszm_GrayLevelVariance					
T2-wavelet-LLL_glszm_GrayLevelVariance					
ADC-wavelet-LLL_glrlm_LongRunHighGrayLevelEmphasis					
**Multivariate model for peripheral zone lesions**					
T2-wavelet-HHH_glszm_GrayLevelVariance T2-wavelet-LLL_glszm_GrayLevelVariance ADC-wavelet-LLL_gldm_HighGrayLevelEmphasis	86 ± 12	80 ± 19	93 ± 6	70 ± 19	84

ADC: apparent diffusion coefficient; NPV: negative predictive value; PPV: positive predictive value; PSA: prostate-specific antigen; SD: standard deviation.

## Data Availability

The data presented in this study are available on request from the corresponding author.

## Abbreviations

AS	active surveillance
ADC	apparent diffusion coefficient
BPH	benign prostate hyperplasia
csPC	clinically significant prostate cancer
DICOM	Digital Imaging and Communication in Medicine
DWI	diffusion-weighted imaging
ISUP	International Society of Urological Pathology
GS	Gleason score
mpMRI	multiparametric magnetic resonance imaging
PACS	Picture Archiving and Communication System
PI-RADS	Prostate Imaging–Reporting and Data System
PC	prostate cancer
PPV	positive predictive value
PSA	prostate-specific antigen
PZ	peripheral zone
ROI	region of interest
T1W	T1-weighted
T2W	T2-weighted
TZ	transition zone
VOI	volume of interest
